# Histological Outcomes and JAK-STAT Signalling in Ulcerative Colitis Patients Treated with Tofacitinib

**DOI:** 10.1093/ecco-jcc/jjae031

**Published:** 2024-03-20

**Authors:** Sara van Gennep, Ivan C N Fung, Djuna C de Jong, Rishand K Ramkisoen, Esmé Clasquin, Jitteke de Jong, Leonie C S de Vries, Wouter J de Jonge, Krisztina B Gecse, Mark Löwenberg, John C Woolcott, Aart Mookhoek, Geert R D’Haens

**Affiliations:** Amsterdam UMC, Department of Gastroenterology and Hepatology, Amsterdam, The Netherlands; Amsterdam UMC, Tytgat Institute for Liver and Intestinal Research, Amsterdam, The Netherlands; Amsterdam UMC, Department of Gastroenterology and Hepatology, Amsterdam, The Netherlands; Amsterdam UMC, Department of Gastroenterology and Hepatology, Amsterdam, The Netherlands; Amsterdam UMC, Department of Gastroenterology and Hepatology, Amsterdam, The Netherlands; Amsterdam UMC, Department of Gastroenterology and Hepatology, Amsterdam, The Netherlands; Amsterdam UMC, Department of Gastroenterology and Hepatology, Amsterdam, The Netherlands; Amsterdam UMC, Tytgat Institute for Liver and Intestinal Research, Amsterdam, The Netherlands; Amsterdam UMC, Department of Gastroenterology and Hepatology, Amsterdam, The Netherlands; Amsterdam UMC, Department of Gastroenterology and Hepatology, Amsterdam, The Netherlands; Pfizer Inc., Collegeville, PA, USA; University of Bern, Department of Pathology, Institute of Tissue Medicine and Pathology, Bern, Switzerland; Amsterdam UMC, Department of Gastroenterology and Hepatology, Amsterdam, The Netherlands

**Keywords:** Tofacitinib, ulcerative colitis, histology

## Abstract

**Background and aims:**

Histological outcomes and JAK-STAT signalling were assessed in a prospective ulcerative colitis [UC] patient cohort after 8 weeks treatment with tofacitinib, an oral Janus kinase [JAK] inhibitor.

**Methods:**

Forty UC patients received tofacitinib 10 mg twice daily for 8 weeks. Treatment response was defined as histo-endoscopic mucosal improvement [HEMI]. Histological remission was defined as a Robarts Histopathology Index [RHI] ≤3 points and histological response as 50% decrease in RHI. Mucosal expression of JAK1-3, tyrosine kinase 2 [TYK2], and total signal transducer and activator of transcription [STAT] 1-6 were assessed using immunohistochemistry [IHC].

**Results:**

At baseline, the median RHI was 14 (interquartile range [IQR] 10–19). Of 40 [65%] patients, 26 had severe endoscopic disease [endoscopic Mayo score 3] and 31/40 [78%] failed prior anti-tumour necrosis factor [anti-TNF] treatment. At Week 8, 15 patients [38%] had HEMI, 23 patients [58%] histological remission, and 34 [85%] histological response. RHI decreased by a median of 14 points [IQR 9-21] in responders [*p* <0.001] and by 6 points [IQR 0-13] in non-responders [*p* = 0.002]. STAT1, STAT3, and STAT5 expression levels decreased significantly in the whole cohort. Responders had lower Week 8 STAT1 expression levels compared with non-responders [0.2%, IQR 0.1-2.8 vs 4.3%, IQR 1.2-11.9, *p* = 0.001], suggesting more profound STAT1 blockade. A trend of higher baseline JAK2 expression was observed in tofacitinib non-responders [2.7%, IQR 0.1-7.7] compared with responders [0.4%, IQR 0.1-2.1].

**Conclusions:**

Tofacitinib treatment resulted in histological improvement in the majority of UC patients and in a substantial decrease of STAT1, STAT3, and STAT5 expression. HEMI was associated with more profound suppression of STAT1.

## 1. Introduction

Tofacitinib is an oral small molecule Janus kinase [JAK] inhibitor for the treatment of moderate to severe ulcerative colitis [UC]. Tofacitinib binds intracellularly to JAKs (JAK1, JAK2, and JAK3 and tyrosine kinase 2 [TYK2]) and inhibits phosphorylation of signal transducers and activators of proteins [STATs]. Phosphorylated STATs [pSTATs] are the biologically active molecules that migrate into the cell nucleus [in homo- or heterodimers] and activate downstream transcription of inflammatory cytokines. Consequently, inhibition of STAT phosphorylation has anti-inflammatory effects in chronic inflammatory disorders, such as UC and rheumatoid arthritis^.1^

The clinical and endoscopic efficacy of tofacitinib in refractory UC has been previously demonstrated.^2^ However, *in vivo* histological and immunological data in colonic mucosa after tofacitinib treatment are lacking. Documentation of histological healing is essential, even in endoscopically quiescent disease, given the association of persistent neutrophils in the epithelium and/or lamina propria with enhanced risk of clinical relapse and dysplasia.^3–6^ Histological healing is not considered as a formal treatment target in UC, but can be considered in addition to endoscopic remission to represent a deeper level of healing.^7^ In addition to routine histology, exploration of immunological changes in colonic mucosa before and after tofacitinib treatment could improve our understanding of the anti-inflammatory effects of tofacitinib at the mucosal level. Ideally, we could identify biomarkers associated with response to this treatment. In this study, we explored mucosal histological and immunological effects of 8 weeks of tofacitinib treatment in patients with moderate-severe UC.

## 2. Materials and Methods

### 2.1. Trial design and study population

The TOFA-histology study was a single-centre, open-label, exploratory study in which adult patients with moderate to severe ulcerative colitis initiated 10 mg tofacitinib twice daily during at least 8 weeks. Between December 2017 and June 2020, patients were invited to participate in this open-label study when they were between 18 and 65 years of age and had active UC, confirmed by sigmoidoscopy and histopathology at least 4 months prior to inclusion. All eligible patients had a baseline total Mayo score of ≥6 and an endoscopic Mayo score [EMS] of ≥2 before treatment initiation. All patients had failed or were intolerant to at least one of the following treatment agents: oral corticosteroids, thiopurines [azathioprine, mercaptopurine, or thioguanine], and/or anti-tumour necrosis factor [anti-TNF] agents [infliximab, adalimumab, or golimumab]. Patients with a history of more than one episode of herpes zoster or a history of disseminated herpes zoster or herpes simplex virus infection were excluded. Other exclusion criteria included evidence of colonic dysplasia at screening sigmoidoscopy, prior UC-related surgery, and a history of malignancy other than adequately treated non-metastatic basal cell or squamous cell skin carcinoma. Following new European Medicines Agency [EMA] guidelines while the study was ongoing, subjects with a risk factor for thromboembolic events [including pulmonary embolisms and deep venous thrombosis] at baseline were excluded from participation. Permitted concomitant therapies included oral aminosalicylates [at a stable dose within 4 weeks prior to baseline], oral glucocorticosteroids [maximum of 20 mg per day for prednisone and 9 mg per day for budesonide at a stable dose for at least 2 weeks before baseline]. Prohibited concomitant therapies included topical therapies [enemas, suppositories], moderate to potent CYP3A inducers or inhibitors, and other advanced UC therapies [ie, immunomodulators, anti-TNF agents, vedolizumab, and ustekinumab].

### 2.2. Ethical, patient and author approval

This study was approved by the Medical Ethics Committee of the Academic Medical Center [METC 2017_120] and registered in the Dutch Trial Register [NL6520]. All patients signed written informed consent prior to inclusion. All authors had access to the study data and reviewed and approved the final manuscript.

### 2.3. Data collection

**Figure 1:**
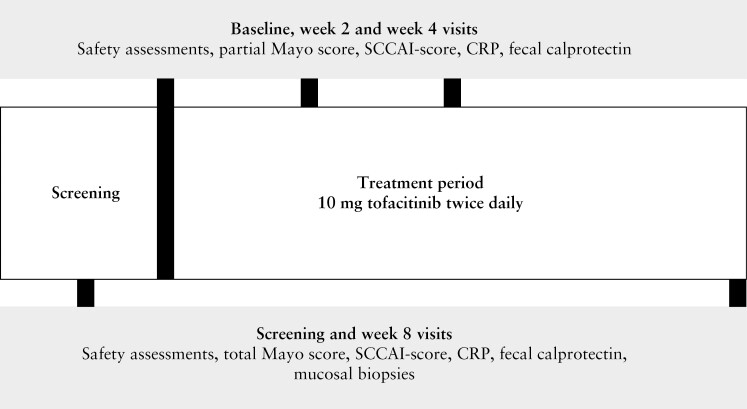
Flowchart of study design.

Included patients underwent six outpatient visits [Figure 1]. At screening and Week 8 [or at early withdrawal] a sigmoidoscopy was performed with biopsy collection (stored in 10% formalin solution and processed for haematoxylin and eosin [H&E] staining). At baseline, two biopsies were collected from the most severely affected area between 15 and 30 cm above the anal verge [or below 15 cm in case of proctitis]. When inflamed mucosa was observed above 30 cm from the anal verge, two additional biopsies were taken from the most inflamed area above 30 cm. After 8 weeks, or at the last visit for patients who did not complete 8 weeks of tofacitinib treatment, biopsies were collected from the same location [in centimetres based on distance from anal verge] as baseline. The total Mayo score was assessed at baseline and after 8 weeks. After 2 and 4 weeks, the partial Mayo score was documented. Adverse events, laboratory test results, concomitant medication, and medication adherence were assessed at each outpatient visit and using patient diaries. Endoscopies were scored for disease severity in each segment by a blinded gastroenterologist using the EMS and the Ulcerative Colitis Endoscopic Index of Severity [UCEIS]. Histological inflammation was quantified by a blinded inflammatory bowel disease [IBD] pathologist [AM] using the RHI and Geboes score [GS].^8,9^ The RHI [range 0–33] is a histological activity grading scale that consists of four items, and the total score is a calculation based on these fout items [Appendix 1].^8^ The GS [continuous score range 0–22] is a histological activity grading scale with seven grade classifications [Appendix 2].^9^ At baseline, the mean RHI and GS were calculated combining the inflamed biopsies [including biopsies taken from the most severely inflamed areas in both sigmoid and descending colon]. After 8 weeks or at the last visit for patients who did not complete 8 weeks of tofacitinib treatment, the mean RHI and GS were calculated by combining the biopsies that were taken at the same location as baseline.

### 2.4. Endpoints

The primary endpoint of the TOFA histology study was the decrease in mucosal inflammatory infiltrate after 8 weeks of treatment with tofacitinib [10 mg twice daily] comparing the average RHI at baseline [mean RHI of all inflamed biopsies available at baseline] with the average RHI [mean RHI of all biopsies that were taken from the same site compared with baseline] at Week 8 or at the last visit for patients who did not complete 8 weeks of tofacitinib treatment, in responders and non-responders. Responders were defined as patients with histo-endoscopic mucosal improvement [HEMI] at 8 weeks. HEMI was defined as an EMS ≤1 and RHI ≤3 points without mucosal neutrophils. Non-responders were defined as patients without HEMI after 8 weeks. Patients who did not complete 8 weeks of tofacitinib treatment were also considered non-responders. A secondary endpoint was the change in GS that was calculated by subtracting the average GS at baseline [mean GS of all inflamed biopsies available] from the average GS at Week 8 or at the last visit for patients who did not complete 8 weeks of tofacitinib treatment [mean GS of all biopsies that were taken from the same site compared with baseline] in both responders and non-responders. Other secondary clinical, endoscopic, and histological outcomes at Week 8 were presented as the proportion of patients that achieved the endpoint after 8 weeks of treatment. Clinical and biochemical improvement over time was assessed at Weeks 2, 4, and 8 using the partial Mayo score [excluding the EMS], C-reactive protein [CRP], and faecal calprotectin [FCP].

### 2.5. Immunohistochemistry [IHC] analysis

Formalin-fixed, paraffin-embedded biopsies, collected from the most severely inflamed segment between 15 and 30 cm above the anal verge [or <15 cm in case of proctitis] at baseline and after 8 weeks, were processed routinely and immunohistochemical staining was performed using the following antibodies: CD3 [rabbit, polyclonal, Dako], CD4 [rabbit, SP35, Abcam], CD8 [rabbit, SP16, Spring Bioscience], CD68 [mouse, KP1, Dako], FOXP3 [mouse, 236A/E7, Invitrogen], MPO [rabbit, polyclonal, Dako], JAK1 [rabbit, 6G4, Cell Signaling], JAK2 [rabbit, D2E12, Cell Signaling], JAK3 [rabbit, polyclonal, Atlas antibodies], TYK2 [rabbit, polyclonal, Abcam], total STAT1 [rabbit, polyclonal, Atlas antibodies], total STAT2 [rabbit, polyclonal, Atlas antibodies], total STAT3 [mouse, 124H6, Cell Signaling], total STAT4 [rabbit, 2H9L5, Invitrogen], total STAT5 [mouse, ERP16671-40, Abcam], and total STAT6 [rabbit, YE361, Abcam]. JAK1-3, TYK2, and total STAT1-6 were validated using different tissue types [ie, skin, tonsil, and breast cancer] and tested on colon tissue before the trial slides were stained. IHC slides were scanned using Philips IntelliSite UltraFast Scanner [Philips, Best, The Netherlands] and uploaded in QuPath v0.3.2.^10^ Pixel width and pixel height were set to 0.25 µm, based on Philips Scanner measurements. Scripts were created through Workflow tab by the following steps: first, biopsies were annotated accurately with the wand tool. Second, QuPath positive cell detection was used to determine the number of positive [DAB-stained] cells to fully automate cell count. Detection image was set to Optical density [OD] sum. To account for differences in staining intensity of the specific antibodies, two different scripts were used for the analysis: OD max script for FOXP3, STAT1, STAT4 and STAT5, and OD mean for CD3, CD4, CD8, CD68, MPO, JAK1, JAK2, TYK2, STAT2, STAT3, and STAT6. Intensity parameters threshold was decreased to 0.05 and maximum background intensity was increased to 5. Intensity threshold parameters score compartment were selected for both OD mean and OD maximum scripts: threshold 1 + was set on 0.15, threshold 2 + was set on 0.175, and threshold 3 + was set on 0.2. The selected annotations were run afterwards with script editor in Automate section. IHC results were reported as the ratios of positive cells in the whole slide image and were calculated as follows: sum of positive cells [including threshold 2 + and 3 + cells] divided by the total number of cells [all cells in the biopsy]. Changes in the abundance of macrophages [CD68], neutrophils [MPO], and T-cell subsets [CD4, CD4, CD8, and FOXP3], as well as JAK1-3, TYK2, and total STAT1-6 expression, were evaluated before and after 8 weeks of treatment and compared between responders and non-responders. In an additional exploratory analysis, we divided the non-responder group into true non-responders [patients without HEMI at Week 8 who stopped tofacitinib at <1 year] and partial responders [patients without HEMI at Week 8 who continued tofacitinib at >1 year].

### 2.6. Statistical analysis

Data was analysed according to an intention-to-treat principle, including all patients who were enrolled in the TOFA-histology trial and received at least one dose of tofacitinib. In case of missing histological, clinical, or endoscopic outcomes, this patient was considered non-responder. Missing IHC data were omitted and the analyses were performed using the remaining data [listwise deletion]. Outcome data were described using means with standard deviations [SD] for normally distributed numerical data, medians with interquartile ranges [IQR] for non-normally distributed numerical data, and numbers with percentages for categorical data. Dichotomous variables were analysed for different outcomes using a chi square test/Fisher’s exact test, and continuous variables were analysed using an unpaired t test or Mann–Whitney U test [depending on distribution]. Wilcoxon signed rank tests were used to compare non-normally distributed scores measured at two different time points. Using Pearson’s correlation testing, associations were assessed between JAK-STAT signalling and RHI. The strength of association was classified using the correlation coefficient [r] value that ranged between 0.1 to 0.3 and -0.1 to -0.3 [weak], 0.3 to 0.7 and -0.3 to -0.7 [moderate], and 0.7 to 1.0 and -0.7 to -1.0 [strong]. Predictors of tofacitinib response were identified using logistic regression analyses. Predictors were identified by including predictors with a *p*-value ≤0.1 in univariable analysis into multivariable analysis using forward selection. Outcomes were presented with odds ratio [OR] and associated 95% confidence interval [CI]. A two-sided *p*-value <0.05 was considered statistically significant. All statistical analyses were performed using SPSS version 26 [release 26.0.0.1].

## 3. Results

### 3.1. Patient population

In total, 52 patients were screened and 40 patients were included between January 2018 and June 2020 [Table 1]. Most patients had at least left-sided UC [95%] and severe endoscopic disease [EMS of 3] at inclusion [65%]. Most included patients had failed anti-TNF [78%] or vedolizumab [55%], and 17 out of 40 patients [43%] had failed both agents prior to inclusion. Median baseline RHI and GS were 14 [IQR 10-19] and 16 [IQR 14-19], respectively. Fifteen patients [38%] were responders and 25 patients [63%] were non-responders at 8 weeks. Of the 25 non-responders, 13 patients [52%] continued treatment over 1 year [considered partial responders] and 12 patients [48%] were true non-responders [stopped tofacitinib within 1 year]. Baseline RHI and GS were comparable between responders and non-responders (15 [9–22] vs 14 [10–18], *p* = 0.889, and 16 [14–20] vs 17 [14–19], *p* = 0.614). Overall, patient and disease characteristics at baseline were comparable between responders and non-responders [Table 1]. A higher percentage of patients in the responder group used concomitant prednisone at baseline [67% vs 16%]. Patients in the non-responder group expressed a higher CRP level at baseline compared with patients in the responder group (10 [1–26] vs 3 [1–6]).

**Table 1:** Baseline characteristics.

	Total	Responders	Non-responders	*p*-value
	*n* = 40	*n* = 15	n = 25	
Male, *n* [%]	15 [38]	7 [47]	8 [32]	0.354
Age [years], median [IQR]	43 [27-55]	42 [32-55]	29 [26-55]	0.727
BMI, median [IQR]	24 [21-28]	26 [22-29]	23 [21-27]	0.102
Active smoking, *n* [%]	3 [8]	2 [13]	1 [4]	0.545
Disease duration [years], median [IQR]	7 [4-12]	9 [4-14]	7 [3-11]	0.501
Prior anti-TNF use, *n* [%]	31 [78]	11 [73]	20 [80]	0.625
Prior vedolizumab use, *n* [%]	18 [45]	9 [60]	9 [36]	0.140
Montreal classification:				0.291
Proctitis [E1], *n* [%]	2 [5]	1 [7]	1 [4]	
Left-sided colitis [E2], *n* [%]^a^	21 [53]	10 [67]	11 [44]	
Pancolitis [E3], *n* [%]	17 [43]	4 [27]	13 [57]	
Concomitant prednisone use, *n* [%]	14 [35]	10 [67]	4 [16]	0.001
Prednisone dosage [mg/day], median [IQR]	15 [10-20]	15 [10-20]	13 [10-23]	0.771
Concomitant budesonide use, *n* [%]	8 [20]	1 [7]	7 [28]	0.219
Budesonide dosage [mg/day], median [IQR]	9 [9-9]	-	9 [9-9]	-
Concomitant mesalazine use, *n* [%]	21 [53]	10 [67]	11 [44]	0.165
Mesalazine dosage [mg/day], median [IQR]	3200 [2700-4400]	3000 [2300-4000]	4000 [3000-4800]	0.235
Baseline TMS, median [IQR]	9 [9-11]	9 [8-11]	10 [9-11]	0.154
Baseline EMS of 3, *n* [%]	26 [65]	7 [47]	19 [76]	0.060
Baseline CRP [mg/L], median [IQR]	6 [I2-18]	3 [1-6]	10 [1-26]	0.021
Baseline FCP [mg/kg], median [IQR]	2263 [562-4495]	2165 [988-4300]	2447 [168-5079]	0.696
Baseline GS [range 0-22], median [IQR]	16 [14-19]	16 [14-20]	17 [14-19]	0.614
Baseline RHI [range 0-33], median [IQR]	14 [10-19]	15 [9-22]	14 [10-18]	0.889

Anti-TNF, anti-tumour necrosis factor; BMI, body mass index; CRP, C-reactive protein; EMS, endoscopic Mayo score; GS, Geboes score [range 0–22]; FCP, faecal calprotectin; IQR, interquartile range; RHI, Robarts Histopathology Index; SD, standard deviation; TMS, total Mayo score.

^a^Upper limit of disease was not visualised in nine patients with left-sided disease, indicating that a number of these patients might had extensive disease at baseline.

### 3.2. Histological outcomes

**Figure 2:**
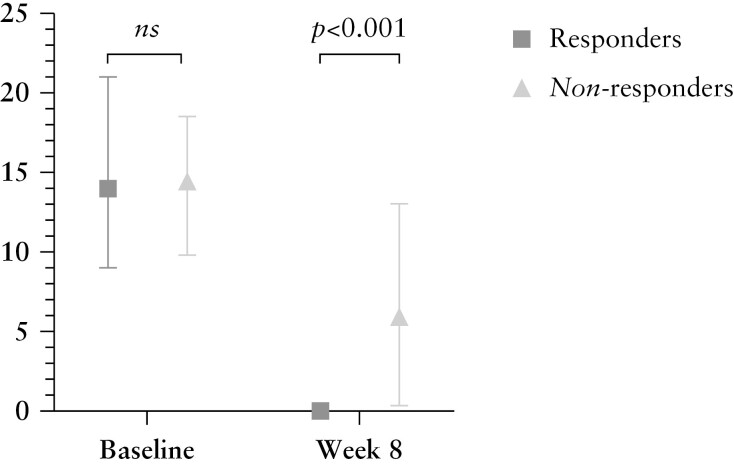
Histological inflammation before and after tofacitinib treatment comparing responders and non-responders. Ns, non-significant; RHI, Robarts Histopathology Index.

Histological remission was attained in 23 patients [58%] after 8 weeks of tofacitinib treatment [Table 2]. Histological response was observed in 34 out of 40 patients [85%] and HEMI [response] in 15 patients [38%]. At 8 weeks, responders to tofacitinib had a significantly lower RHI compared with non-responders (0 [0–0] vs 6 [0–13], *p* <0.001, Figure 2). In addition, the decrease in RHI from baseline to Week 8 was significantly more pronounced in responders compared with non-responders (Δ–14 [-21 to -9] vs Δ–6 [-10 to -8], *p* = 0.002). Likewise, a more substantial decrease in RHI was observed for responders compared with non-responders including only biopsies that were taken from 30 cm below the anal margin (Δ–22 [-27 to -12] vs Δ–6 [-21 to -1], *p* = 0.001). No significant difference was found for responders versus non-responders solely including biopsies taken above 30 cm above the anal margin (Δ–12 [-17 to -7] vs Δ–9 [-14 to -1], *p* = 0.210). In comparison with RHI, responders had significantly lower GS after 8 weeks of treatment compared with non-responders (1 [0–5] vs 11 [7–15], *p *<0.001), and the decline in GS was more evident in responders compared with non-responders after 8 weeks of treatment (Δ–14 [-16 to -13] vs Δ–5 [-11 to 0], *p* <0.001).

**Table 2:** Endpoint definitions and outcomes after 8 weeks of tofacitinib treatment.

Endpoints	Definition	*N* [%]
Clinical response	Decreased TMS ≥3 and ≥30% compared with baseline, with an accompanying decrease in the subscore for rectal bleeding of ≥1 or an absolute rectal bleeding subscore ≤1	15 [38%]
Clinical remission	TMS ≤2, with no individual subscore >1, and rectal bleeding subscore = 0	10 [25%]
Symptomatic remission	TMS ≤2, with no individual subscore >1, and rectal bleeding subscore = 0 and stool frequency subscore = 0	5 [13%]
Endoscopic response	Decreased EMS ≥1 compared with baseline	23 [58%]
Endoscopic improvement	EMS ≤1	20 [50%]
Endoscopic remission	EMS = 0	8 [20%]
Deep remission	TMS ≤2, with no individual subscore > 1 and EMS = 0, and rectal bleeding subscore = 0	7 [18%]
Histological response	50% reduction in average RHI at Week 8 compared with baseline or histological remission at Week 8 [including all inflamed biopsies available]	34 [85%]
Histological remission	Absence of neutrophils in the lamina propria, absence of crypt destruction, and absence of erosion and ulcers [RHI ≤3, with RHI subscore ‘neutrophils in epithelium’ = 0 and ‘erosion or ulcer’ = 0] assessed in all inflamed biopsies available during the Week 8 sigmoidoscopy	23 [58%]
Histo-endoscopic mucosal improvement [HEMI]	Endoscopic improvement and histological remission	15 [38%]
Deep clinical, endoscopic, and histological remission	Clinical, endoscopic, and histological remission and rectal bleeding subscore of 0	6 [15%]

EMS, endoscopic Mayo score; RHI, Robarts Histopathology Index; TMS, total Mayo score.

### 3.3. Clinical, biochemical, and endoscopic outcomes

#### 3.3.1. Clinical and biochemical outcomes

After 8 weeks of treatment, 10 patients [25%] were in clinical remission and 15 out of 40 patients [38%] had clinical response. Clinical disease activity measured using the partial Mayo score [PMS, including only rectal bleeding, stool frequency, and physician global assessment] decreased significantly after 8 weeks compared with baseline in both responders (2 [1–2] vs 7 [6–8], *p* <0.001) as well as in non-responders (3 [1–7] vs 7 [6–8], *p *<0.001). A significant decline in PMS was already observed after 2 weeks both in responders (3 [2–4] vs 7 [6–8], *p* <0.001) and non-responders (4 [3–7] vs 7 [6–8], *p* <0.001]. The magnitude of decline in PMS was not different comparing responders with non-responders after 2, 4, and 8 weeks, respectively. Responders started with substantially higher baseline CRP levels compared with non-responders to tofacitinib (3 [1–6] vs 10 [1–26], *p* = 0.021). Yet, a significant decline in CRP was observed after 8 weeks both in in responders (3 [1–6] vs 4 [0–8] mg/L, *p *= 0.002) and non-responders (6 [1–11] vs 12 [2–26] mg/L, *p* = 0.029). A substantial decrease was found after 2 weeks of therapy both in responders (0 [0–1] vs 3 [1–6] mg/L, *p* = 0.009) and non-responders (3 [1–11] vs 12 [2–26] mg/L, *p* = 0.029]. In addition, the magnitude of decrease was comparable between responders and non-responders after 2, 4, and 8 weeks, respectively. FCP levels were substantially lower after 8 weeks of tofacitinib treatment in responders (51 [13–81] vs 2165 [988–4300] mg/kg, *p* = 0.001) and non-responders (1204 [243–2694] vs 1964 [257–5309], *p* = 0.040). A significant decline was noticed after 2 weeks of treatment in responders (264 [137–891] vs 2165 [988–4300] mg/kg, *p* = 0.002), but not in non-responders (2952 [586–4759] vs 1964 [257–5309] mg/kg, *p* = 0.876).

#### 3.3.2. Endoscopic and combined outcomes

In total, 20 out of 40 patients [50%] had endoscopic improvement after 8 weeks of treatment with tofacitinib. Deep clinical, endoscopic, and histological remission was observed in six patients [15%] after 8 weeks tofacitinib.

### 3.4. Immunohistochemistry [IHC]

#### 3.4.1. Macrophages [CD68], neutrophils [MPO], and T cell subsets [CD3, CD4, CD8, and FOXP3]

In responders, treatment with tofacitinib led to a significant reduction in all mucosal immune cell populations under study, including T cells (CD3: 32.0% [7.7–13.0] vs 19.2% [3.0–7.2], *p* = 0.021; CD4: 21.0% [9.6–24.8] vs 9.2% [6.4–13.7], *p* = 0.041; CD8: 5.3% [4.2–7.0] vs 3.0% [1.6–4.2], *p* = 0.019; FOXP3: 3.2% [1.9–4.8] vs 1.6% [0.7–2.1], *p* = 0.026]) granulocytes including neutrophils (MPO: 3.8% [1.8–4.6] vs 0.1% [0.0–0.8], *p* = 0.028) and macrophages (CD68: 6.5% [3.1–10.0] vs 2.2% [1.9–4.1], *p* = 0.008). A decline of approximately the same magnitude in CD68 and MPO were observed in non-responders (7.8% [3.9–11.8] vs 4.4% [2.5–8.6], *p = *0.11 and4.6% [1.8–13.7] vs 1.7% [0.3–5.6], *p = *0.005, respectively].

#### 3.4.2. Baseline JAK–STAT expression

**Figure 3:**
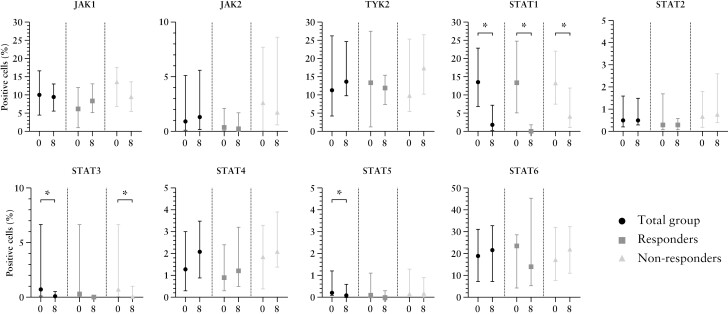
JAK-STAT expression before and after 8 weeks of tofacitinib treatment. X-axis represents the time of measurement [0 = baseline, 8 = Week 8 or at the last visit for patients who did not complete 8 weeks of tofacitinib treatment]. Y-axis represents the percentage positive stained cells. The median percentages of positive stained JAK1, JAK2, and total STAT4 cells were higher in non-responders compared with responders at baseline, yet not statistically significant. Median baseline total STAT6 expression was numerically higher in responders compared with non-responders at baseline, although not statistically significant. A significant decline in total STAT1, STAT3, and STAT5 positive cells was observed in the total group of patients after 8 weeks, without a clear association with histo-endoscopic mucosal improvement [HEMI].

**Figure 4:**
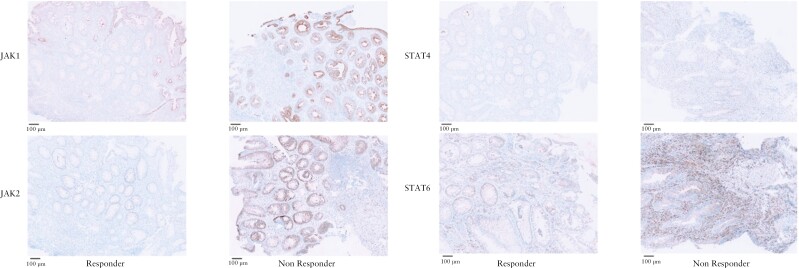
JAK1, JAK2, total STAT4, and total STAT6 expression at baseline in responders and non-responders. A trend towards higher JAK1, JAK2, and total STAT4 expression and lower total STAT6 expression was observed in non-responders compared with responders at baseline.

Although not statistically significant, higher baseline JAK1, JAK2, and total STAT4 levels were found in non-responders (13.7% [6.9–17.6], 2.7% [0.1–7.7], and 1.9% [0.4–3.3]) compared with responders (6.2% [1.1–12.1], 0.4% [0.1–2.1], and 0.9% [0.3–2.4]), [Figure 3 and Figure 4, respectively]. For JAK2, this finding was confirmed in an additional exploratory analysis; true non-responders expressed the highest JAK2 levels (3.3% [0.2–6.8]), lower JAK2 levels (0.9% [0.0–2.8]) were found for partial responders, and the lowest baseline JAK2 levels were found for true responders (0.4% [0.0–2.1]). Baseline total STAT6 expression was non-significantly higher in responders (23.6% [4.2–28.8]) compared with non-responders (17.4% [7.6–32.1]).

#### 3.4.3. Changes in JAK–STAT expression levels after 8 weeks of tofacitinib treatment

**Figure 5:**
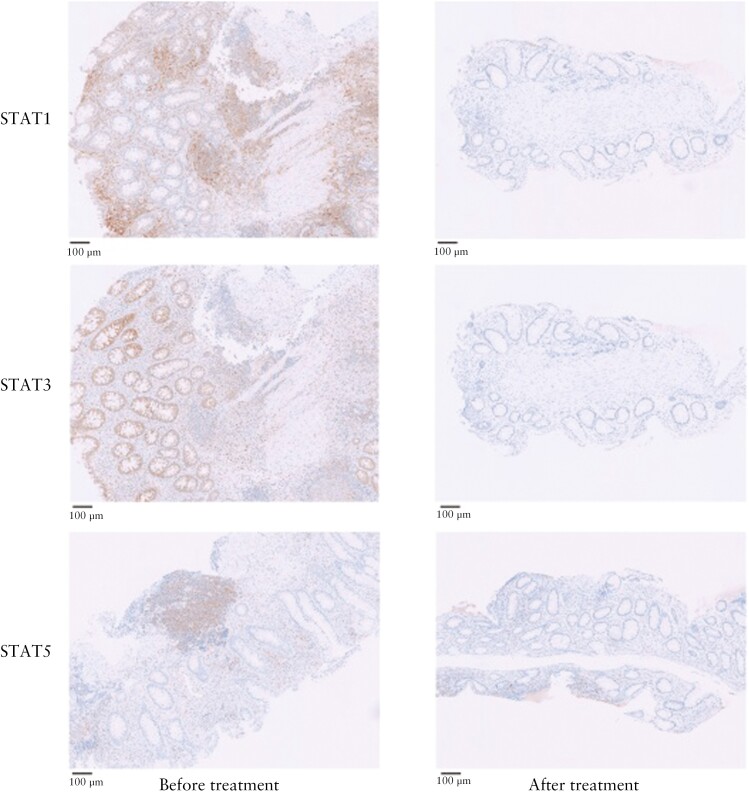
Total STAT1, STAT3, and STAT5 expression at baseline and after 8 weeks of tofacitinib. A significant reduction in total STAT1, STAT3, and STAT5 expression was observed following tofacitinib treatment in the total cohort.

A significant reduction in total STAT1 (13.5% [6.9–22.8] vs 1.8% [0.3–7.2], *p* <0.001), STAT3 (0.7% [0.1–6.7] vs 0.1% [0.0–0.5], *p* = 0.002), and STAT5 (0.2% [0.1–1.2] vs 0.1% [0.0–0.6], *p = *0.026) expression was observed after therapy in the whole population [Figure 5]. For total STAT1, this substantial decline was detected in responders as well as in non-responders, although responders expressed a significantly lower percentage of STAT1 positive cells compared with non-responders after 8 weeks of therapy (0.2% [0.1–2.8] vs 4.3% [1.2–11.9], p = 0.001). This finding was confirmed in an additional exploratory analysis; highest total STAT1 levels (9.5% [3.5–25.8]) were found for true non-responders, lower levels were found for partial non-responders (0.9% [0.0–2.8]), and true responders expressed the lowest STAT1 levels (0.4% [0.0–2.1]) at baseline. Total STAT3 expression was significantly decreased in non-responders, but the decrease was not significant in responders. Subgroup analyses revealed a non-significant total STAT5 reduction after 8 weeks in both tofacitinib responders and non-responders. Comparing responders with non–responders, Week 8 JAK2 (0.2% [0.1–1.7] vs 1.8% [0.6–8.6], *p* = 0.008) and total STAT2 expression (0.3% [0.1–0.6] vs 0.8% [0.4–2.6], *p* = 0.004) were significantly lower, although no significant decrease compared with baseline values was observed. No JAK3-postive cells were present before or after 8 weeks of tofacitinib treatment; therefore we excluded this marker from further analyses.

### 3.5. Correlation between JAK-STAT protein expression and histological inflammation

Using pooled data from both baseline and Week 8 biopsies, a moderate correlation between total STAT1 and RHI was observed [r = 0.506, *p* <0.001]. Weak correlations were observed between total STAT2 and total STAT5 with RHI [r = 0.323, *p = *0.005 and r = 0.320, *p = *0.006, respectively]. JAK1, JAK2, TYK2, and total STAT1, STAT3, STAT4, and STAT6 were not associated with degree of inflammation.

### 3.6. Biological predictors of tofacitinib response

Higher baseline CRP levels were found to be associated with a lower chance of tofacitinib response in univariable analysis (OR 0.9 [0.8–1.0], *p* = 0.025). Another potential predictor associated with tofacitinib response in univariable analysis that was found eligible [*p* ≤0.1] to be included into multivariable analysis was lower baseline JAK2 expression (OR 0.8 [0.6–1.0], *p* = 0.094). Among the two variables included in the multivariable logistic regression analysis, only lower baseline CRP was significantly associated with response after 8 weeks of tofacitinib (OR 0.89 [0.79–0.99], *p* = 0.036).

## 4. Discussion

This is one of the first prospective studies to investigate the impact of tofacitinib 10 mg twice daily [BID] on histological inflammation, and demonstrated a significant decrease in histological inflammation after 8 weeks of tofacitinib 10 mg BID treatment. The novelty of this research and its important findings are amplified when noting that absence of residual microscopic inflammation in UC patients is important, even in endoscopic quiescent disease, given the association of persistent neutrophils in the epithelium and/or lamina propria with an increased risk of clinical relapse and dysplasia/neoplasia.^3–6^

In our cohort, we found a relatively high percentage of histological remission [58%] after 8 weeks of tofacitinib treatment compared with 24% [using the Nancy Index] reported by Verstockt *et al*. after 8 weeks tofacitinib.^11^ In comparison, trials that reported histological remission rates with other therapeutic agents also reported lower percentages: 15% for infliximab, 7–18% for adalimumab, 37% for ustekinumab after 8 weeks [histological remission defined as GS ≤3], and 26% for vedolizumab after 14 weeks [RHI ≤2], respectively.^12–15^ Interestingly, mucosal healing rates for infliximab [45%] and adalimumab [52%] were comparable to endoscopic improvement rates of 50% that we report here [using the same definition of endoscopic Mayo subscore ≤1] after 8 weeks.^12,13^ In a Phase 3 study evaluating effectiveness of ustekinumab in UC, the rate of endoscopic improvement was lower [27%] compared with our study.^15^ The discrepancy in histological remission rates might be explained by the location at which the biopsies were obtained. In our study, biopsies were collected following a strict protocol, from the most severely inflamed area between 15 and 30 cm from the anal verge [or <15 cm if only proctitis was present] at baseline and at the same location [in centimetres] after 8 weeks. In the two previously mentioned anti-TNF studies, biopsies were taken at random locations from the sigmoid and rectum [two biopsies per segment] and histological activity from the worst affected biopsy was recorded.^12,13^ As some patients in our study had proximal endoscopic healing, with remaining endoscopic disease activity in the rectum, this might explain the relative high percentage of patients achieving histological remission.

Using IHC, we found significantly lower percentages of total STAT1, STAT3, and STAT5 after 8 weeks of treatment in the total cohort. Our results reveal JAK-STAT inhibition at a mucosal level in all patients using tofacitinib, without a clear association with treatment response. An interesting finding was that tofacitinib response was associated with significantly lower Week 8 total STAT1 expression, possibly reflecting the degree of inflammation in the biopsies. An association between total STAT1 and RHI outcomes was found. Inhibition of the JAK-STAT signalling pathway affects numerous cytokine pathways, and its role in IBD pathology is very complex. Expression of STAT1, for example, has been reported to be increased in inflamed colonic biopsies of UC patients, and induction of remission using glucocorticoids led to a decrease of phosphorylated STAT1 *in vitro.*^16^ Yet, both pro-inflammatory properties [in lymphocyte populations] and protective features [in macrophages and intestinal epithelial cells] have been attributed to STAT1 activation.^17,18^ Recently, it was shown that phosphorylated STAT3 [pSTAT3] levels were substantially decreased after 8 weeks of tofacitinib treatment in a refractory UC population.^11^ In agreement with our findings, negligible association with treatment outcome was found. However, the authors did find a significant association between lamina propria pSTAT3 and the degree of inflammation. In our study, we found no correlation between total STAT3 and RHI. In contrast to our findings, in a rheumatoid arthritis cohort, tofacitinib did not cause significant suppression of total STAT1 and STAT3 levels in peripheral blood monocytes, T cells, and B cells.^19^ However, they did show that responders to tofacitinib expressed higher baseline pSTAT1, pSTAT3, and pSTAT5 levels in peripheral blood monocytes compared with non-responders to tofacitinib, which was not found for total STAT1 and STAT 3 levels. Differences may exist between serum and mucosal JAK-STAT expression and, as is the case in rheumatoid arthritis, *in vitro* and *in vivo* findings might reveal conflicting results.

A few [non-significant] trends were observed in our findings. A higher percentage JAK1, JAK2, and STAT4 cells and lower percentage of STAT6 cells were found at baseline in non-responders compared with responders. This trend was particularly prominent for baseline JAK2 expression, since we observed highest levels for true non-responders to treatment, lower levels for partial responders, and the lowest levels for tofacitinib responders. This finding was not confirmed for JAK1, total STAT4, and total STAT6. Likewise, Week 8 JAK2 levels were significantly higher in non-responders to tofacitinib. Prior *in vitro* studies have shown only moderate affinity of tofacitinib for JAK2, and it therefore has always been stated that JAK2 only plays a minor role in the efficacy of tofacitinib. Our preliminary results, however, might indicate that patients with higher JAK2 expression at baseline are potentially non-responders to tofacitinib. Another possibility is that they might benefit from higher tofacitinib dosage or a longer treatment duration, since higher mucosal levels of tofacitinib are associated with treatment response, as was recently shown by Verstockt *et al*.^20^

As mentioned earlier, this exploratory study is one of the first prospective studies to assess histological outcomes and JAK-STAT signalling before and after tofacitinib treatment in UC patients. One of its major strengths is the blinded scoring of endoscopic and histological outcomes performed by a dedicated IBD gastroenterologist and a dedicated IBD pathologist, and the use of [combined] validated scoring systems. In addition, mucosal biopsies were collected using a stringent predefined protocol. With application of 16 different antibodies, including JAK and STAT antibodies, this study is also one of the first to describe detailed immunological patterns of the gut mucosa before and after tofacitinib treatment *in vivo*.

However, limitations of this study also need to be acknowledged. First, the number of patients included in this study was rather low. Second, no biopsies were collected below 15 cm above the anal verge when patients had left-sided colitis. Therefore, this might have led to discrepancies between the proportion of patients with endoscopic improvement [which include scoring of the rectum] and histological remission, and thereby introduced higher histological remission rates. Third, in three cases [at baseline], limited material was present to perform histological scoring accurately, and one patient was included who already met the criteria for histological remission at baseline. Although this patient had objectified endoscopic disease of at least moderate severity, this might have introduced bias in our sample. In addition, a few IHC data were missing and were omitted from further evaluation, which might have influenced our results. However, missing cases were divided equally between responders and non-responders to treatment. Last, we did not differentiate between membrane-bound inactive STAT and activated STAT translocated to the nucleus in this study.

In conclusion, treatment with tofacitinib significantly reduces histological inflammation in the colonic mucosa after 8 weeks in patients with moderate to severe UC, and resulted in histological improvement in most patients. The percentage of positive total STAT1, STAT3, and STAT5 cells substantially declined after 8 weeks tofacitinib treatment in the total cohort. This downregulation was observed in both tofacitinib responders and non-responders. Lower total STAT1 expression at 8 weeks was associated with treatment response, probably reflecting degree of mucosal inflammation. Non-significant trends towards higher baseline JAK1, JAK2, and total STAT4 expression and lower baseline total STAT6 expression were observed in non-responders to tofacitinib. High baseline JAK2 expression is potentially associated with non-response to tofacitinib. Our findings pave the way for further research to find biomarkers to predict [sustained] response to tofacitinib in UC. Future studies should include JAK and [p]STAT levels, pSTAT/STAT ratios, and tofacitinib [tissue] exposure levels in colonic mucosa and ideally combine these data with peripheral blood assays. Ideally, these studies should be performed in a blinded, randomised, controlled trial using short-term and long-term outcomes.

## Supplementary Data

Supplementary data are available at *ECCO-JCC* online.

jjae031_suppl_Supplementary_Tables_1-4_Appendix_1-2

## Data Availability

The data underlying this article will be shared on reasonable request to the corresponding author.
