# Supplementary material for: Histological Outcomes and JAK-STAT Signalling in Ulcerative Colitis Patients Treated with Tofacitinib
Source: J Crohns Colitis. 2024 Mar 20;18(8):1283–91. doi: 10.1093/ecco-jcc/jjae031 (PMC11324337; doi:10.1093/ecco-jcc/jjae031)
Supplement: jjae031_suppl_Supplementary_Tables_1-4_Appendix_1-2 [file jjae031_suppl_supplementary_tables_1-4_appendix_1-2.docx]

**APPENDIX 1 Robarts Histopathology Index (RHI)**

|  | | | |
| --- | --- | --- | --- |
| **Grade** | **Description** | **score** | |
| **Chronic inflammatory infiltrate** | | | |
| 0 | No increase | | 0 |
| 1 | Mild but unequivocal increase | | 1 |
| 2 | Moderate increase | | 2 |
| 3 | Marked increase | | 3 |
| **Lamina propria neutrophils** | | | |
| 0 | No increase | | 0 |
| 1 | Mild but unequivocal increase | | 1 |
| 2 | Moderate increase | | 2 |
| 3 | Marked increase | | 3 |
| **Neutrophils in epithelium** | | | |
| 0 | None | | 0 |
| 1 | <5% crypt involved | | 1 |
| 2 | <50% crypt involved | | 2 |
| 3 | >50% crypt involved | | 3 |
| **Erosion or ulceration** | | | |
| 0 | No erosion, ulceration or granulation tissue | | 0 |
| 1 | Recovering epithelium plus adjacent inflammation | | 1 |
| 1 | Probable erosion-focally stripped | | 1 |
| 2 | Unequivocal erosion | | 2 |
| 3 | Ulcer or granulation tissue | | 3 |
| **RHI (range 0-33)** = 1 × chronic inflammatory cell infiltrate level [4 levels] + 2 × lamina propria neutrophils [4 levels] + 3 × epithelial neutrophils [4 levels] + 5 × erosion or ulceration [4 levels]. | | | |

**APPENDIX 2. Geboes Score (GS)**

|  | | | |
| --- | --- | --- | --- |
| **Grade** | **Description** | **Continuous GS** | |
| **Structural (architectural change)** | | | |
| 0 | No abnormality | | 0 |
| 0.1 | Mild abnormality | | 1 |
| 0.2 | Mild or moderate diffuse or multifocal abnormalities | | 2 |
| 0.3 | Severe diffuse or multifocal abnormalities | | 3 |
| **Chronic inflammatory infiltrate** | | | |
| 1 | No increase | | 3 |
| 1.1 | Mild but unequivocal increase | | 4 |
| 1.2 | Moderate increase | | 5 |
| 1.3 | Marked increase | | 6 |
| **Lamina propria eosinophils** | | | |
| 2A.0 | No increase | | 6 |
| 2A.1 | Mild but unequivocal increase | | 7 |
| 2A.2 | Moderate increase | | 8 |
| 2A.3 | Marked increase | | 9 |
| **Lamina propria neutrophils** | | | |
| 2B.0 | No increase | | 9 |
| 2B.1 | Mild but unequivocal increase | | 10 |
| 2B.2 | Moderate increase | | 11 |
| 2B.3 | Marked increase | | 12 |
| **Neutrophils in epithelium** | | | |
| 3 | None | | 12 |
| 3.1 | <5% crypt involved | | 13 |
| 3.2 | <50% crypt involved | | 14 |
| 3.3 | >50% crypt involved | | 15 |
| **Crypt destruction** | | | |
| 4 | None | | 15 |
| 4.1 | Probable-local excess of neutrophils in part of crypt | | 16 |
| 4.2 | Probable-marked attenuation | | 17 |
| 4.3 | Unequivocal crypt destruction | | 18 |
| **Erosion or ulceration** | | | |
| 5 | No erosion, ulceration or granulation tissue | | 18 |
| 5.1 | Recovering epithelium plus adjacent inflammation | | 19 |
| 5.2 | Probable erosion-focally stripped | | 20 |
| 5.3 | Unequivocal erosion | | 21 |
| 5.4 | Ulcer or granulation tissue | | 22 |
| **GS (range 0-22)** = The most severe lesion was scored. | | | |

**SUPPLEMENTARY MATERIAL**

## **SUPPLEMENTARY TABLE 1. Change in histological scores from baseline to week 8 in responders and *non-*responders.**

|  | **Response** | ***Non-*response** | ***p-*value** |
| --- | --- | --- | --- |
| **ΔRHI (total)***, median [IQR]* | -14 [-21 to -9] | -6 [-10 to -1] | 0.002 |
| **ΔRHI (biopsies <30 cm)***, median [IQR]* | -22 [-27 to -12] | -6 [-21 to -1] | 0.001 |
| **ΔRHI (biopsies >30 cm)***, median [IQR]* | -12 [-17 to -7] | -9 [-14 to -1] | 0.210 |
| **ΔGS (total)***, median [IQR]* | -14 [-21 to -9] | -5 [-11 to 0] | <0.001 |
| **ΔGS (biopsies <30 cm)***, median [IQR]* | -21 [-21 to -15] | -8 [-18 to -2] | <0.001 |
| **ΔGS (biopsies >30 cm)***, median [IQR]* | -14 [-17 to -13] | -12 [-16 to -3] | 0.084 |
| *GS, Geboes Score; cm, centimeter; IQR, interquartile range; RHI, Robarts Histopathology Index* | | | |

**SUPPLEMENTARY TABLE 2. Change in clinical and biochemical outcomes after 8 weeks of tofacitinib treatment in responders and *non-*responders.**

|  | **Response** | ***Non-*response** | ***p-*value** |
| --- | --- | --- | --- |
| **ΔPMS (week 2)***, median [IQR]* | -3 [-5 to -1] | -3 [-4 to 0] | 0.415 |
| **ΔPMS (week 4)***, median [IQR]* | -4 [-5 to -3] | -3 [-5 to -1] | 0.258 |
| **ΔPMS (week 8)***, median [IQR]* | -5 [-6 to -4] | -3 [-6 to -1] | 0.058 |
| **ΔCRP (week 2)***, median [IQR]* | -2 [-4 to 0] | -4 [-11 to 0] | 0.185 |
| **ΔCRP (week 4)***, median [IQR]* | -2 [-3 to 0] | -5 [-18 to 0] | 0.110 |
| **ΔCRP (week 8)***, median [IQR]* | -2 [-4 to 0] | -4 [-10 to 0] | 0.720 |
| **ΔFCP (week 2)***, median [IQR]* | -2246 [-3382 to -538] | 0 [-951 to 2831] | 0.016 |
| **ΔFCP (week 4)***, median [IQR]* | -1092 [-4445 to -120] | -14 [-1103 to 826] | 0.017 |
| **ΔFCP (week 8)***, median [IQR]* | -2254 [-4433 to -760] | -289 [-2574 to 112] | 0.089 |
| *CRP, C-reactive protein; FCP, fecal calprotectin; IQR, interquartile range; PMS, partial Mayo score* | | | |

## **SUPPLEMENTARY TABLE 3. Percentage CD3, CD4, CD8, CD68, FOXP3 and MPO positive cells before and after 8 weeks tofacitinib treatment**

|  | **Total group,** *N*=40  **Responders**, *N*=15  **Non-responders,** *N*=25 | | | | | |
| --- | --- | --- | --- | --- | --- | --- |
|  | **CD3** | **CD4** | **CD8** | **CD68** | **FOXP3** | **MPO** |
| **Baseline** |  |  |  |  |  |  |
| ***N (total group)*** | **31** | **38** | **39** | **37** | **37** | **37** |
| **Median (%)** | **18.1** | **17.8** | **4.5** | **6.9** | **3.8** | **4.0** |
| **IQR** | **11.4-22.1** | **9.6-24.8** | **3.1-5.8** | **3.9-10.9** | **2.7-4.9** | **1.9-11.3** |
| *N (responders)* | 12 | 14 | 14 | 15 | 14 | 13 |
| Median (%) | 32.0 | 21.0 | 5.3 | 6.5 | 3.2 | 3.8 |
| IQR | 7.7-13.0 | 9.6-24.8 | 4.2-7.0 | 3.1-10.0 | 1.9-4.8 | 1.8-4.6 |
| *N (non-responders)* | 19 | 24 | 25 | 22 | 23 | 24 |
| Median (%) | 16.5 | 16.0 | 3.5 | 7.8 | 3.9 | 4.6 |
| IQR | 10.8-22.1 | 8.7-26.3 | 2.8-5.4 | 3.9-11.8 | 3.3-5.0 | 1.8-13.7 |
| **Week 8** |  |  |  |  |  |  |
| ***N (total group)*** | **37** | **39** | **40** | **38** | **39** | **37** |
| **Median (%)** | **11.9** | **11.0** | **3.3** | **3.8** | **2.2** | **0.7** |
| **IQR** | **8.0-16.0** | **7.9-16.8** | **1.9-5.8** | **2.2-6.5** | **1.4-4.1** | **0.1-5.0** |
| *N (responders)* | 14 | 15 | 15 | 14 | 15 | 14 |
| Median (%) | 19.2 | 9.2 | 3.0 | 2.2 | 1.6 | 0.1 |
| IQR | 3.0-7.2 | 6.4-13.7 | 1.6-4.2 | 1.9-4.1 | 0.7-2.1 | 0.0-0.8 |
| *N (non-responders)* | 23 | 24 | 25 | 24 | 24 | 23 |
| Median (%) | 14.7 | 15.1 | 3.6 | 4.4 | 3.2 | 1.7 |
| IQR | 9.8-21.3 | 8.3-20.2 | 2.5-6.5 | 2.5-8.6 | 1.9-5.4 | 0.3-5.6 |
| ***Z-statistic (total group)*** | **-2.195** | **-1.697** | **-1.842** | **-3.669** | **-2.325** | **-3.582** |
| ***p-value (total group)*** | **0.028***** | **0.090** | **0.065** | **<0.001***** | **0.020***** | **<0.001***** |
| *Z-statistic (responders)* | -2.312 | -2.040 | -2.354 | -2.668 | -2.229 | -2.197 |
| *p-value (responders)* | 0.021*** | 0.041*** | 0.019*** | 0.008*** | 0.026*** | 0.028*** |
| *Z-statistic (non-responders)* | -0.762 | -0.365 | -0.471 | -2.555 | -1.055 | -2.808 |
| *p-value (non-responders)* | 0.446 | 0.715 | 0.638 | 0.011* | 0.291 | 0.005* |
| *N, number of patients; IQR, interquartile range; * statistically significant* | | | | | | |

## **SUPPLEMENTARY TABLE 4. Percentage JAK-STAT positive cells before and after 8 weeks tofacitinib treatment**

|  | **Total group,** *N*=40  **Responders**, *N*=15  **Non-responders,** *N*=25 | | | | | | | | |  |
| --- | --- | --- | --- | --- | --- | --- | --- | --- | --- | --- |
|  | **JAK1** | **JAK2** | **TYK2** | **STAT1** | **STAT2** | **STAT3** | **STAT4** | **STAT5** | **STAT6** |  |
| **Baseline** |  |  |  |  |  |  |  |  |  |  |
| ***N (total group)*** | **32** | **38** | **40** | **38** | **38** | **38** | **38** | **38** | **40** |  |
| **Median (%)** | **10.1** | **0.9** | **11.4** | **13.5** | **0.5** | **0.7** | **1.3** | **0.2** | **19.1** |  |
| **IQR** | **4.5-16.7** | **0.1-5.1** | **4.3-26.3** | **6.9-22.8** | **0.2-1.6** | **0.1-6.7** | **0.3-3.0** | **0.1-1.2** | **7.1-31.3** |  |
| *N (responders)* | 11 | 13 | 15 | 14 | 14 | 14 | 14 | 15 | 15 |  |
| Median (%) | 6.2 | 0.4 | 13.3 | 13.0 | 0.3 | 0.3 | 0.9 | 0.1 | 23.6 |  |
| IQR | 1.1-12.1 | 0.1-2.1 | 1.3-27.6 | 5.2-24.9 | 0.1-1.7 | 0.0-6.7 | 0.3-2.4 | 0.0-1.1 | 4.2-28.8 |  |
| *N (non-responders)* | 21 | 25 | 25 | 24 | 24 | 24 | 24 | 23 | 25 |  |
| Median (%) | 13.7 | 2.7 | 10.0 | 13.5 | 0.7 | 0.8 | 1.9 | 0.2 | 17.4 |  |
| IQR | 6.9-17.6 | 0.1-7.7 | 5.5-25.4 | 7.5-22.0 | 0.2-1.8 | 0.1-6.6 | 0.4-3.3 | 0.2-1.3 | 7.6-32.1 |  |
| **Week 8** |  |  |  |  |  |  |  |  |  |  |
| ***N (total group)*** | **33** | **38** | **40** | **38** | **40** | **38** | **37** | **38** | **40** |  |
| **Median (%)** | **9.5** | **1.3** | **13.7** | **1.8** | **0.5** | **0.1** | **2.1** | **0.1** | **21.7** |  |
| **IQR** | **5.6-13.2** | **0.2-5.6** | **9.9-24.6** | **0.3-7.2** | **0.3-1.5** | **0.0-0.5** | **0.9-3.5** | **0.0-0.6** | **7.2-32.7** |  |
| *N (responders)* | 12 | 13 | 15 | 13 | 15 | 14 | 14 | 13 | 15 |  |
| Median (%) | 8.4 | 0.2 | 11.9 | 0.2 | 0.3 | 0.0 | 1.2 | 0.0 | 14.1 |  |
| IQR | 5.2-13.1 | 0.1-1.7 | 7.4-15.4 | 0.1-1.8 | 0.1-0.6 | 0.0-0.2 | 0.5-3.2 | 0.0-0.3 | 5.3-45.7 |  |
| *N (non-responders)* | 21 | 25 | 25 | 25 | 25 | 24 | 23 | 25 | 25 |  |
| Median (%) | 9.7 | 1.8 | 17.5 | 4.3 | 0.8 | 0.1 | 2.1 | 0.2 | 22.1 |  |
| IQR | 5.6-13.6 | 0.6-8.6 | 10.3-26.6 | 1.2-11.9 | 0.4-2.6 | 0.0-1.0 | 1.4-3.9 | 0.0-0.9 | 10.9-32.5 |  |
| ***Z-statistic (total group)*** | **-0.165** | **-0.083** | **-0.914** | **-4.619** | **-0.718** | **-3.158** | **-0.943** | **-2.231** | **-0.538** |  |
| ***p-value (total group)*** | **0.869** | **0.934** | **0.361** | **<0.001*** | **0.473** | **0.002*** | **0.346** | **0.026*** | **0.591** |  |
| *Z-statistic (responders)* | -0.700 | -1.490 | -0.114 | -2.824 | -0.471 | -1.712 | -0.471 | -1.083 | -0.511 |  |
| *p-value (responders)* | 0.484 | 0.136 | 0.910 | 0.005* | 0.638 | 0.087 | 0.638 | 0.279 | 0.609 |  |
| *Z-statistic (non-responders)* | -0.631 | -0.848 | -1.117 | -3.514 | -1.314 | -2.646 | -0.666 | -1.947 | -0.390 |  |
| *p-value (non-responders)* | 0.528 | 0.397 | 0.264 | <0.001* | 0.189 | 0.008* | 0.506 | 0.052 | 0.696 |  |
| *N, number of patients; IQR, interquartile range; * statistically significant* | | | | | | | | | | |
